# Time to haematologist visit and non-haematological referral from primary health care during blood cancer diagnosis – findings from a large national survey in Australia

**DOI:** 10.1186/s12885-026-15757-1

**Published:** 2026-02-17

**Authors:** Mohammad Radwanur Talukder, Emily Kovacev, Bill Stavreski, Sarah DeLacey

**Affiliations:** 1 Health Services, Leukaemia Foundation, Adelaide, Australia; 2 Global Health and Migration Unit, Department of Women’s and Children’s Health, Uppsala University, Uppsala, Sweden; 3 Present Address: Women and Kids Theme, South Australian Health and Medical Research Institute, Adelaide, Australia

**Keywords:** Blood cancer, Australia, Primary health care, Time interval, Referral, Awareness, Leukaemia, Lymphoma, Multiple myeloma, Myelodysplastic neoplasms, Myeloproliferative neoplasms

## Abstract

**Background:**

Blood cancers are the third most common cancer in Australia. However, little is known about their diagnostic pathway in the Australian context. This research aimed to assess the interval between first primary health care (PHC) contact and first haematologist visit, and referrals to specialists other than haematologists (non-haematological referrals) during blood cancer diagnoses.

**Methods:**

An online national survey was conducted with patients living with blood cancers, such as leukaemia, lymphoma, multiple myeloma, myeloproliferative neoplasms (MPN) and myelodysplastic neoplasms (MDS) between October and November 2022. Univariate and adjusted logistic regression models were applied to examine sociodemographic factors (age, sex, income, residence location, country of birth, private health insurance) and type of blood cancers associated with extended PHC-haematologist visit intervals (> 1-month) and non-haematological referrals.

**Results:**

Of the total 1758 participants, 26.3% (95% CI 24.3, 28.4%) patients experienced an extended PHC-haematologist visit interval (> 1-month) and of 1749 participants, 24.8% (95% CI 22.8, 26.9%) patients experienced non-haematological referrals during their diagnosis stage. Women were more likely to report an extended PHC-haematologist visit interval (adjusted odds ratio aOR 1.38, 95% CI 1.10, 1.73) or non-haematological referrals (aOR 1.42, 95%CI 1.12, 1.80). Regional Australians (aOR 1.37, 95%CI 1.08, 1.73) were more likely to experience an extended PHC -haematologist visit interval, but the odds of non-haematological referrals was not significant for them. Being older (≥ 65 years) (aOR 0.59, 95% CI 0.36, 0.97) and overseas-born (aOR 0.73, 95%CI 0.54, 0.99) were associated with a lower likelihood of non-haematological referrals. Private insurance holders (aOR 1.19, 95% CI 1.05, 1.35) were also more likely to have specialist referrals other than haematologists. Patients with an indolent type of blood cancers, such as lymphomas, multiple myeloma and MDS, are more likely to experience an extended PHC-haematologist visit interval or non-haematological referrals.

**Conclusions:**

A multi-pronged approach, including raising awareness of blood cancer signs and symptoms among both patients and health care professionals and improving access to GPs and specialist services, particularly in regional and rural Australia, is required to improve timely help-seeking during diagnosis. More evidence is also needed on clusters of non-specific symptoms indicative of haematological cancers underpinning guidelines and optimal care pathways to support possible use of automated software prompts and prompt diagnosis.

**Supplementary Information:**

The online version contains supplementary material available at 10.1186/s12885-026-15757-1.

## Background

Blood cancers (including leukaemia, lymphoma, multiple myeloma, myelodysplastic neoplasms and myeloproliferative neoplasms) are a group of cancers that affect bone marrow and blood cells [1, 2] and pose a significant public health burden in Australia and globally [2, 3]. Between 1990 and 2021/22, globally, Australasia was one of the regions with the highest increasing trend of age-standardised incidence rates for most blood cancers [3]. In Australia, when combined, blood cancers are the third most common cancer, with currently over 150,000 people living with blood cancer, and the second leading cause of cancer death [4]. By 2035, the prevalence of blood cancer is estimated to double in Australia, with an economic cost of $71.9 billion for this growing problem [5].

This growing burden of blood cancer is compounded by unique challenges of blood cancer that are non-screenable or non-preventable through known lifestyle or medical interventions, such as healthy diet, exercise, vaccinations or drugs (e.g. statins for lowering risk of high cholesterol). In general, patient delay and health system delays, including those in primary and tertiary health care, have been reported in cancer care [6, 7]. The non-specific nature and non-serious interpretation of symptoms, which are often considered a normal state of well-being are common contributing factors among patients and healthcare professionals, leading to delays in help-seeking and diagnosis [8]. A recent literature review reported several patient demographic factors (being women, young etc.) and health system factors (non-acute presentations, longer waiting time for specialist appointment etc.) contributing to delays in blood cancer diagnosis [8]. Multiple visits to primary health care doctors before diagnosis are also common for people living with blood cancer [9, 10]. Delayed diagnosis and presentation with blood cancer are associated with fewer treatment options and increased morbidity risk (e.g. anaemia, higher rates of renal failure, fractures, spinal cord compression in multiple myeloma and advanced disease at presentation) [11, 12]. Moreover, delayed diagnosis is associated with decreased quality of life, increased hospitalisations and higher healthcare costs and significant psychosocial impact [12]. Although no single diagnostic delay threshold associated with harm for blood cancers is available, evidence suggests delays beyond approximately 2–3 months and further have been associated with advanced disease features, organ damage, and higher complication rates, particularly in multiple myeloma and lymphoma [11, 13]. Reducing such delays in their care-seeking pathway for blood cancer is likely to lead to improved outcomes and quality of life [8, 14, 15]. However, little is known about factors associated with primary health care (PHC) to haematologist visit intervals and referrals from PHC to specialists other than haematologists during blood cancer diagnosis in Australia.

To tackle the growing burden of blood cancer, the Australian Research Roadmap for Blood Cancer 2024 has highlighted the importance of identifying ways to prevent and detect blood cancer early [16]. Although the Australian blood cancer optimal care pathways (OCPs) recommend a timeframe for GPs to support timely diagnosis and referral of suspected blood cancer cases [17], the current state of blood cancer diagnosis pathway is unknown. Knowledge of the diagnostic time intervals and journey for blood cancers in Australia will, therefore, be pertinent to addressing individual and system-level issues and improving timely help-seeking behaviours and diagnostic evaluation according to the recommendations of OCPs.

## Methods

An online survey was conducted with patients living with leukaemia, lymphoma, multiple myeloma, myeloproliferative neoplasms (MPN), myelodysplastic neoplasms (MDS) or amyloidosis in Australia between October and November 2022 as part of the State of the Nation (SoTN): Blood Cancers in Australia report. The survey was representative of sub-types, Australian states and territories, regional status, age and private health insurance status. The participants, who were known to be alive before administration of survey, were invited to participate via direct emails. A flyer with a QR code was also shared within the Blood Cancer Taskforce network (https://www.leukaemia.org.au/research-and-advocacy/advocacy-and-policy/blood-cancer-taskforce/) in Australia to promote recruitment. Participation in the survey was voluntary. A total of 4600 blood cancer patients or patients with blood disorders and/or their carers, including patients diagnosed with blood cancer before or after 2018, completed the survey. Some preliminary descriptive results from the survey were published in the SoTN 2022 report [4]. In this paper, we examined the factors associated with an extended time interval between first presentation to PHC with symptoms and first haematologist consultation and non-haematological referrals during blood cancer diagnosis journey in Australia, self-reported by blood cancer patients. Study population comprised patients aged 15 years and above who had a new diagnosis of blood cancer between 2018 and the survey start. To keep the findings relevant for blood cancer, we restricted our analysis to established blood cancer conditions – leukaemia, lymphoma, multiple myeloma, MDS and MPN. The survey questionnaire is available via the published report [4]. A waiver of individual informed consent for secondary analysis of survey data was approved by the GWHREC (HREC 2024/ETH02670). All data analysis was conducted in a de-identified way.

### Outcome measure

Time to first appointment with haematologists from primary health care (PHC) presentation was determined from the patients’ reported time between their first presentation to a GP with symptoms and their first appointment with a haematologist [6]. Studies varied in their measurement of the time interval and used different time points to define the delay in blood cancer diagnosis, such as time from first symptom to first presentation in primary care, time to referral to specialists and time to diagnosis [8]. Antel et al. (2019) reported diagnostic delay as > 6 weeks of health practitioner interval (i.e. first contact to diagnostic biopsy) [11]. The Australian Optimal Care Pathways (OCPs) for different blood cancers suggest a 2–4 week timeframe from GP presentation to first haematologist consults and diagnostic workups for suspected blood cancers, except for acute leukaemias or presentations related to rapidly progressive symptoms that require immediate management [17]. According to the UK National Institute for Health and Care Excellence (NICE) guideline Suspected cancer: recognition and referral (NG12) [18], patients presenting with symptoms suggestive of haematological malignancy (e.g., unexplained pallor, fatigue, fever, lymphadenopathy, splenomegaly, or bleeding) should have initial investigations such as a full blood count within 48 h, and those with suggestive positive findings should be referred on a suspected cancer pathway (typically within the 2-week / Faster Diagnosis Standard diagnostic timeframe) for specialist assessment. Therefore, in this study, we categorised the interval between first PHC contact and haematologist visit into less than a month and more than a month (i.e. extended PHC-haematologist visit interval) in bivariate analysis. Moreover, we also examined the factors associated with first referrals to specialists other than haematologists (i.e. non-haematological referral).

### Covariates

Data on socio-demographic conditions (age, sex, income, residence location, country of birth, private health insurance etc.) and type of blood cancers were applied as independent variables. For statistical robustness, acute lymphoblastic leukaemia and acute myeloid leukaemia were combined into acute leukaemia. Acute leukaemia also included acute promyelocytic leukaemia and mixed phenotype acute leukaemia. Similarly, chronic lymphocytic leukaemia and chronic myeloid leukaemia were combined as chronic leukaemia. Chronic leukaemia also included hairy cell leukaemia. Lymphoma was categorised into Hodgkin and Non-Hodgkin lymphoma. Non-Hodgkin Lymphoma (NHL) included all relevant sub-types, e.g. Burkitt’s lymphoma, follicular lymphoma, Mantle cell lymphoma, marginal lymphoma, Waldenström’s macroglobulinaemia, aggressive B cell lymphoma, T cell lymphoblastic lymphoma, CNS lymphoma, grey zone lymphoma. Essential thrombocythaemia, polycythaemia rubra vera and myelofibrosis were grouped into MPN. Place of residence was recorded as metropolitan or regional for each state and territory except for Australian Capital Territory (ACT), Northern Territory (NT) and Tasmania (TAS), and all of them combined categorised into metro vs. regional. According to Australian Modified Monash Model 7 (MMM7) classification NT and TAS were classified into regional only and ACT was classified into metro only [19]. Country of birth was categorised as Australia vs. overseas.

### Statistical analysis

Descriptive statistics (frequency distribution, percentage) were applied to report the socio-demographic characteristics of the participants. Pearson’s chi-square test was applied to compare the distribution of each explanatory variable for extended PHC-haematologist visit interval and non-haematological referrals. A *p*-value of < 0.05 was considered statistically significant. To identify the socio-demographic risk factors associated with the extended PHC-haematological visit interval and non-haematological referrals, separate logistic regression analysis model was used. First, univariate analysis was conducted to examine the association between each of the socio-demographic factors (age, sex, income, residence location, country of birth, private health insurance etc.) and type of blood cancers [8] with the outcomes of interest (i.e. extended PHC-haematological visit interval and non-haematological referrals). Subsequently, all the variables were included simultaneously in the adjusted model. Moreover, we also performed separate adjusted models for the Australian states and territories (Australian Capital Territory (ACT), New South Wales (NSW), Queensland (QLD), South Australia (SA), Tasmania (TAS), Victoria (VIC) and Western Australia (WA)) as a category replacing the metro-regional category and stratified analysis for NSW for each of the outcomes. The strength of the association was measured using the odds ratio (OR) and 95% confidence intervals. STATA version 14.2 was used for data analysis.

## Results

Out of the total 4600 participants who completed the survey, after excluding those diagnosed before 2018, participants aged 14 years and below, blood disorder cases (e.g. amyloidosis) (*n* = 96), those missing or unable to recall the time interval (*n* = 54), and those missing or unable to recall referral information (*n* = 63), 1758 cases were included for the PHC-haematologist visit interval analysis and 1749 cases for the non-haematological referral analysis. Table 1 presents the socio-demographic and clinical characteristics of participants reporting extended interval between first PHC contact and haematologist first visit or non-haematological referrals during their blood cancer diagnosis. Of the total 1758 participants, 26.3% (95% confidence interval (CI) 24.3, 28.4%) reported an extended time (i.e. > 1 month) interval from their GP presentation to first visit to haematologist during their blood cancer diagnosis journey (Table 1). It is also noteworthy that, among the less than 1-month group, 26.4% patients were diagnosed incidentally or through suspicion of other conditions. This figure is also consistent with previous research [14]. 29% (95% CI 26.2, 32.0%) of female patients, while 23.0% (95% CI 20.2, 26.1%) of male patients living with blood cancer, experienced an extended time (*p* = 0.004). Similarly, a significantly higher proportion of regional Australians diagnosed with blood cancer (28.6%, 95% CI 26.0, 31.4%), compared to metro residents (22.7%, 95% CI 19.7, 26.0%), experienced an extended time interval (Table 1).

**Table 1:** Socio-demographic and clinical characteristics of participants by time to haematologist visit category and referral to a haematologist category from primary health care (PHC)

Variables	First PHC presentation to haematologist visit interval				PHC referral to a haematologist			
	< 1-month	> 1-month	Total	*p* value	Referral direct to haematologist	Referral to specialists other than haematologists	Total	*p* value
All cases (N)	1295	463	1758		1315	434	1749	
% (95% CI)	73.7 (71.6, 75.7)	26.3 (24.3, 28.4)			75.2 (73.1, 77.2)	24.8 (22.8, 26.9)		
Sex (*n* = 1751)					*n* = 1743			
Women (n)	672	275	947	0.004	704	268	948	0.003
% (95% CI)	71.0 (68.0, 73.8)	29.0 (26.2, 32.0)			72.4 (69.4, 75.1)	27.6 (24.9, 30.6)		
Men (n)	619	185	804		624	171	795	
% (95% CI)	77.0 (73.9, 79.8)	23.0 (20.2, 26.1)			78.5 (75.5, 81.2)	21.5 (18.8, 24.5)		
Age groups in years (*n* = 1755)					*n* = 1746			
15–34 (n)	92	28	120	0.513	82	37	119	0.254
% (95% CI)	76.7 (68.2, 83.4)	23.3 (16.6, 31.8)			68.9 (60.0, 76.6)	31.1 (23.4, 40.0)		
35–64 (n)	616	213	829		619	201	820	
% (95% CI)	74.3 (71.2, 77.2)	25.7 (22.8, 28.8)			75.5 (72.4, 78.3)	24.5 (21.7, 27.6)		
>=65 (n)	584	222	806		612	195	807	
% (95% CI)	72.5 (69.3, 75.4)	27.5 (24.6, 30.7)			75.8 (72.8, 78.7)	24.2 (21.3, 27.2)		
Residence (*n* = 1750)					*n* = 1741			
Metro (n)	522	153	675	0.006	523	152	675	0.078
% (95% CI)	77.3 (74.0, 80.3)	22.7 (19.7, 26.0)			77.5 (74.2, 80.5)	22.5 (19.5, 25.8)		
Regional (n)	767	308	1075		786	280	1086	
% (95% CI)	71.4 (68.6, 74.0)	28.6 (26.0, 31.4)			73.7 (71.0, 76.3)	26.3 (23.7, 29.0)		
Country of birth (*n* = 1755)					*n* = 1746			
Australia (n)	1024	362	1386	0.627	1016	357	1373	0.019
% (95% CI)	73.9 (71.5, 76.1)	26.1 (23.9, 28.5)			74.0 (71.6, 76.2)	26.0 (23.7, 28.4)		
Overseas (n)	268	101	369		298	75	373	
% (95% CI)	72.6 (67.8, 76.9)	27.4 (23.1, 32.2)			79.9 (75.5, 83.7)	20.1 (16.3, 24.5)		
Income (*n* = 1758)					*n* = 1749			
< $50k (n)	596	232	828	0.214	622	201	823	0.021
% (95% CI)	72.0 (68.8, 74.9)	28.0 (25.1, 31.2)			75.6 (72.5, 78.4)	24.4 (21.6, 27.5)		
>=$50k (n)	488	168	646		466	179	645	
% (95% CI)	74.3 (70.8, 77.5)	25.7 (22.5, 29.2)			72.3 (68.7, 75.6)	27.7 (24.4, 31.3)		
Undisclosed (n)	224	66	284		227	54	281	
% (95% CI)	77.1 (71.8, 81.6)	22.9 (18.4, 28.1)			80.8 (75.7, 85.0)	19.2 (15.0, 24.3)		
Private insurance (*n* = 1726)					*n* = 1716			
No (n)	496	189	685	0.324	524	152	676	0.058
% (95% CI)	72.4 (68.9, 75.6)	27.6 (24.4, 31.1)			77.5 (74.2, 80.5)	22.5 (19.5, 25.8)		
Yes (n)	776	265	1041		764	276	1040	
% (95% CI)	74.5 (71.8, 77.1)	25.5 (22.9, 28.2)			73.5 (70.7, 76.1)	26.5 (23.9, 29.3)		
Blood cancer^ diagnosis	*n* = 1758				*n* = 1749			
Acute Leukaemia (n)	359	53	412	< 0.001	352	56	408	< 0.001
% (95% CI)	87.1 (83.5, 90.0)	12.9 (10.0, 16.5)			86.3 (82.6, 89.3)	13.7 (10.7, 17.4)		
Chronic Leukaemia (n)	171	76	247		215	33	248	
% (95% CI)	69.2 (63.2, 74.7)	30.8 (25.3, 36.8)			86.7 (81.8, 90.3)	13.3 (9.6, 18.2)		
Hodgkin Lymphoma (n)	61	36	97		61	31	92	
% (95% CI)	62.9 (52.8, 71.9)	37.1 (28.1, 47.2)			66.3 (56.0, 75.2)	33.7 (24.7, 44.0)		
MDS (n)	68	22	90		70	21	91	
% (95% CI)	75.6 (65.6, 83.4)	24.4 (16.6, 34.4)			76.9 (67.1, 84.5)	23.1 (15.5, 32.9)		
MM (n)	234	88	322		243	79	322	
% (95% CI)	72.7 (67.5, 77.3)	27.3 (22.7, 32.5)			75.5 (70.5, 79.9)	24.5 (20.1, 29.5)		
MPN (n)	60	28	88		72	17	89	
% (95% CI)	68.2 (57.7, 77.1)	31.8 (22.9, 42.3)			80.9 (71.3, 87.8)	19.1 (12.2, 28.7)		
NHL (n)	342	160	502		303	197	500	
% (95% CI)	68.1 (63.9, 72.1)	31.9 (27.9, 36.1)			60.6 (56.2, 64.8)	39.4 (35.2, 43.8)		

^ blood cancer – *MDS* Myelodysplastic neoplasm, *MM* Multiple myeloma, *MPN* Myeloproliferative neoplasm, *NHL* Non-Hodgkin Lymphoma

When examined by states and territories, including both metro and regional locations of Australia, the highest proportion for extended PHC-haematologist visit interval was reported by Northern Territory patients (47.4%, 95% CI 26.3, 69.4%). However, the sample size was small (*n* = 19) as observed in the wider confidence interval (Supplementary Table 1). One-third of patients, each from Australian Capital Territory (ACT) and Western Australia, reported extended PHC-haematologist visit interval (Supplementary Table 1). The proportion of patients reported extended PHC-haematologist visit interval in New South Wales (NSW), Queensland (QLD), South Australia (SA), Tasmania (TAS) and Victoria (VIC) ranged from 23.7 to 28.1% (Supplementary Table 1). However, this difference was not statistically significant (*p* = 0.386).

By blood cancer subtype, the highest proportion of patients with Hodgkin lymphoma (37.1%, 95% CI 28.1, 47.2%) experienced an extended time for the first consultation with a haematologist. More than one-third of patients with either NHL or MPN or chronic leukaemia experienced an extended time interval. No significant difference in proportion for extended PHC-haematologist visit interval was observed among patients by their age, income, country of birth or private health insurance status (Table 1).

Higher proportions of patients who experienced non-haematological referrals (*N* = 1749) during their blood cancer diagnosis journey were women (27.6%, 95% CI 24.9, 30.6% vs. males 21.5%, 95% CI 18.8, 24.5%) and Australia-born patients (26.0%, 95% CI 23.7, 28.4% vs. overseas-born 20.1%, 95% CI 16.3, 24.5%) and these differences were statistically significant (Table 1). Although a higher proportion of regional residents (26.3%, 95% CI 23.7, 29.0%) reported non-haematological referrals than metro residents (22.5%, 95% CI 19.5, 25.8%), but it was not statistically significant (*p* = 0.078). In contrast to extended PHC-haematologist visit interval, the proportion of patients experiencing non-haematological referrals was lowest in NT (16.7%) followed by ACT (18.6%). Patients in SA (30.8%) followed by VIC (30.3%) reported highest proportions of non-haematological referrals (Supplementary Table 1).

### Factors associated with extended PHC-haematologist visit interval

In the adjusted analysis, women (aOR 1.38, 95%CI 1.10, 1.73) compared to male blood cancer patients, regional Australians (aOR 1.37, 95% CI 1.08, 1.73) compared to metro residents were more likely to experience an extended time interval from their first GP presentation to their first visit with a haematologist. Patients of all types of blood cancers, compared to those with acute leukaemia, were more likely to experience an extended PHC-haematologist visit interval (Table 2).

**Table 2:** Multivariable logistic regression analysis on factors associated with extended PHC-haematologist visit interval in Australia

	Unadjusted			Adjusted		
	Odds Ratio	95% Confidence Interval	*p* value	Odds Ratio	95% Confidence Interval	*p* value
Age groups in years						
15–34	Ref			Ref		
35–64	1.13	0.72, 1.8	0.579	1.12	0.68, 1.86	0.647
>=65	1.25	0.80, 1.96	0.333	1.24	0.74, 2.08	0.413
Sex						
Men	Ref			Ref		
Women	1.37	1.10, 1.70	0.004	1.38	1.10, 1.73	0.006
Residence						
Metro	Ref			Ref		
Regional	1.37	1.10, 1.71	0.006	1.37	1.08, 1.73	0.009
Country of birth						
Australia	Ref			Ref		
Overseas	1.07	0.82, 1.38	0.627	1.09	0.83, 1.43	0.548
Income						
< $50k	Ref			Ref		
>=$50k	0.89	0.70, 1.12	0.319	0.98	0.75, 1.27	0.854
Undisclosed	0.76	0.56, 1.04	0.092	0.76	0.54, 1.06	0.103
Private insurance						
No	Ref			Ref		
Yes	0.95	0.85, 1.05	0.324	0.97	0.86, 1.09	0.597
Blood cancer^ diagnosis						
Acute Leukaemia	Ref			Ref		
Chronic Leukaemia	3.01	2.03, 4.47	0.000	2.91	1.93, 4.37	0.000
Hodgkin Lymphoma	4.00	2.42, 6.61	0.000	3.75	2.21, 6.34	0.000
MDS	2.19	1.25, 3.84	0.006	2.27	1.28, 4.04	0.005
MM	2.55	1.74, 3.72	0.000	2.51	1.69, 3.71	0.000
MPN	3.16	1.85, 5.39	0.000	3.10	1.79, 5.37	0.000
NHL	3.17	2.25, 4.47	0.000	3.10	2.18, 4.42	0.000

^ blood cancer – *MDS* Myelodysplastic neoplasm, *MM* Multiple myeloma, *MPN* Myeloproliferative neoplasm, *NHL* Non-Hodgkin Lymphoma

A separate adjusted logistic regression model, using state and territories as a category replacing the metro-regional category, revealed a similar direction of associations for factors of extended PHC-haematologist visit interval, except there were no significant associations with any separate states and territories (Supplementary Table 2). Stratified analysis of NSW data also revealed a similar direction of associations in the adjusted model, including regional NSW patients more likely to experience an extended PHC-haematologist visit interval (aOR 1.95, 95%CI 1.19, 3.20). However, the association between women and extended time interval was strengthened but remained non-significant in the adjusted model for NSW data (Supplementary Table 2).

### Factors associated with PHC referrals to specialists other than haematologists

In the adjusted model (Table 3), being women (aOR 1.42, 95% CI: 1.12,1.80) and privately insured (aOR 1.19, 95% CI: 1.05,1.35) were each associated with a higher likelihood of experiencing non-haematological referrals in the blood cancer diagnostic journey. However, in the adjusted model, there was no evidence of an association between regional areas and referral to a non-haematological specialist. (Table 3). In contrast, being older (≥ 65 years) (aOR 0.59, 95% CI: 0.36,0.97) and overseas-born (aOR 0.73, 95% CI: 0.54,0.99) were associated with a lower likelihood. Patients with Hodgkin or NHL, MDS, or multiple myeloma were most likely to be referred to other specialists before seeing a haematologist.

**Table 3:** Multivariable logistic regression analysis on factors associated with PHC referral to specialists other than haematologists during blood cancer diagnosis in Australia

	Unadjusted			Adjusted		
	Odds Ratio	95% Confidence Interval	*p* value	Odds Ratio	95% Confidence Interval	*p* value
Age groups in years						
15–34	Ref			Ref		
35–64	0.72	0.47,1.09	0.124	0.62	0.38,1.00	0.049
>=65	0.71	0.46,1.07	0.105	0.59	0.36,0.97	0.037
Sex						
Men	Ref			Ref		
Women	1.39	1.12,1.74	0.003	1.42	1.12,1.80	0.004
Residence						
Metro	Ref			Ref		
Regional	1.23	0.98,1.54	0.078	1.21	0.95,1.54	0.131
Country of birth						
Australia	Ref			Ref		
Overseas	0.72	0.54,0.95	0.02	0.73	0.54,0.99	0.04
Income						
< $50k	Ref			Ref		
>=$50k	1.19	0.94,1.50	0.149	1.22	0.93,1.60	0.151
Undisclosed	0.74	0.52,1.03	0.075	0.72	0.50,1.04	0.083
Private insurance						
No	Ref			Ref		
Yes	1.24	0.99,1.56	0.058	1.19	1.05,1.35	0.008
Blood cancer^ diagnosis						
Acute Leukaemia	Ref			Ref		
Chronic Leukaemia	0.97	0.61,1.54	0.895	0.93	0.58,1.50	0.777
Hodgkin Lymphoma	3.19	1.91,5.35	0.000	3.16	1.84,5.41	0.000
MDS	1.89	1.07,3.31	0.027	2.06	1.14,3.70	0.017
MM	2.05	1.40,3.00	0.000	2.29	1.53,3.40	0.000
MPN	1.48	0.82,2.70	0.196	1.39	0.74,2.59	0.306
NHL	4.09	2.93,5.71	0.000	4.45	3.14,6.31	0.000

^ blood cancer – *MDS* Myelodysplastic neoplasm, *MM* Multiple myeloma, *MPN* Myeloproliferative neoplasm, *NHL* Non-Hodgkin Lymphoma

Like extended PHC-haematologist visit interval, similar directions of associations for factors of non-haematological referrals were observed applying state and territories category in the model (Supplementary Table 3). Stratified analysis of NSW data only showed significant associations between non-haematological referrals and women, and for Hodgkin or NHL or MM, indicating factors of non-haematological referrals are variables across the Australian states and territories (supplementary Table 3).

## Discussion

Our research is one of the few studies in Australia that show factors associated with an extended time interval from first presentation to PHC to first haematologist consultation and primary health care referrals to specialists other than haematologists, self-reported by patients with lived experience during their blood cancer diagnosis journey. 26% of Australians with lived experience reported - an extended interval between first PHC contact and haematologist visit and 25% experienced referral to specialists other than haematologists during their blood cancer diagnosis journey. Women were more likely to experience an extended PHC-haematologist visit interval and non-haematological referrals. Moreover, being regional and having private health insurance were more likely to be associated with referrals to specialists other than haematologists. In contrast, overseas-born and older Australians were less likely to be referred to specialists other than haematologists.

Quantitative research on examining time intervals during blood cancer diagnoses has been scarce, and none were available from Australia [8]. Inconsistent quantitative methods in those studies, in terms of data collection (e.g. self-reported survey, medical records, medical insurance claim dates) and variation in defining and measuring time intervals, make cross-study comparisons for such outcomes difficult [7]. However, findings from qualitative studies revealed that lower cancer suspicion by both patients and HCPs was a unifying reason for diagnostic delay [8].

Our finding of a higher likelihood of experiencing extended PHC-haematologist visit interval by women is consistent with previous research with the diagnostic interval defined as from the date of first help-seeking to diagnosis [14, 20]. Women more frequently than men are likely to receive other differential diagnoses than cancer, whose somatic presentations are often vague or non-specific, potentially delaying diagnosis [21]. Such an appraisal of symptoms may also result in referrals to specialists other than haematologists. In general, like with other chronic conditions and cancers, women have also reported worse experiences along their cancer care pathway [22, 23]. This gender difference in care experience could be due to women being more critical of their care [22], as they value psychosocial support from health care team more than their counterparts [24].

Although the diagnostic delay for other cancers among regional residents has been reported [25, 26], our research also indicates an extended interval PHC-haematologist visits for rural and regional patients before they are diagnosed with blood cancer. Urban and rural disparity in healthcare accessibility and outcomes is a well-documented and persistent issue in Australia [27]. Limited access to GPs, availability of specialised services, longer hospital waiting times and delays and difficulties accessing specialist appointments in regional and rural areas [27] are likely to contribute to an extended time interval. In Australia, regional centres (109.9), small rural towns (78.2) and remote communities (68.1) have fewer GPs compared to metropolitan areas (115.2 FTE per 100,000 population) to deliver primary care services [28]. Lack of time or resources for longer travel, cost of GP appointments due to lower availability of bulk billing, and higher socioeconomic disadvantage in regional and rural areas could all contribute to this extended time interval [29, 30]. Continuity of care with the same GP is also less frequent in the regional and rural Australian context [30]. Studies have reported potential delay in PHC for blood cancer diagnosis associated with locum consultations [8]. While overall blood cancer patients from regional Australia did not report a significantly increased risk of non-haematological referrals, the factors associated with non-haematological referrals appeared to vary by states and territories, as observed through the stratified analysis of NSW data. Non-significant increased risk of non-haematological referrals is rather more likely because of limited access to any specialist care close to their locations [31]. Future research should consider examining state- and territory-wide variations in cancer care pathways and their impact on the diagnosis and treatment of cancer cases in Australia. Concerted efforts between government and non-government health services and professional peak bodies are required to ensure equitable access to HCPs, more support for GPs and their professional development curriculum is consistent with the location-specific disease burden, particularly HCPs who are serving in the rural and regional towns.

Like previous research, we also observed extended PHC-haematologist visit interval and referral to specialists other than haematologists for lymphomas and multiple myeloma compared to acute leukaemias [8, 9, 20]. Qualitative research with multiple myeloma patients revealed referral to a different specialty other than haematology by GP before diagnosis [9]. A negative blood result following the first GP consultation for a suspected lymphoma case also contributes to the delay in referral to a haematologist and diagnosis [8], highlighting the importance of follow-up. While patients with chronic leukaemias and MPN experienced extended PHC-haematologist visit interval, we did not observe any increased risk of non-haematological referrals for these two conditions. The asymptomatic nature of these blood cancers often makes their diagnosis incidental from blood tests at routine health checks or check-ups for other comorbidities [14]. Although international guidelines [32, 33] and OCPs [17] recommend immediate referral to tertiary care for further evaluation, risk stratification and ensuring appropriate treatment modalities of acute leukaemias, in this research, 13% of patients with acute leukaemias either experienced a delay from PHC to haematology visit or non-haematological referral. In both acute and chronic leukaemias timely diagnosis and assessment are critical for reducing complications, symptoms, identifying appropriate treatment options and improving survival [34].

Although we did not observe a significantly extended PHC-haematologist visit interval among young Australians, they are likely to experience referrals to specialists other than haematologists indicates that HCPs’ less awareness and suspicion of blood cancer and related symptoms in patients during their young age. This is also consistent with current literature [8]. Higher referrals to specialists other than haematologists for patients with private health insurance are likely to be either due to increased access to specialists or their ability to access specialists privately without long waiting periods seen in the public system, or to source a second opinion. Multiple visits to GP before being diagnosed with blood cancer have been reported elsewhere [9, 35]. However, improved awareness of primary HCPs on blood cancer symptoms across different age groups is required to address the issue of referrals to specialists other than haematologists.

Unlike some cancers, the clinical presentation, particularly initial symptoms, of blood cancers is generally recognised as being broad and ill-defined, which may be nonspecific and difficult to differentiate from those of benign, self-limiting conditions [8]. However, frequently reported symptoms, by a large cohort of leukaemia, lymphoma and myeloma patients (*n* = 3329) from the UK’s Haematological Research Network, were tiredness, pain, lump, shortness of breath/cough, skin problems, abnormal sweating and infections, while two-thirds of the patients reported either tiredness, pain or lump [14]. In a matched case-control study with primary health care data from UK, lymphadenopathy alone or in combination with either fatigue, shortness of breath or infection showed a PPV of > 10% for NHL in patients ≥ 60 years [36]. However, individual symptoms of myeloma in primary care generally showed a low risk (PPVs < 1%), although risk estimates increased for some symptoms when combined with leucopenia or hypercalcaemia (PPVs > 10%) [37]. The UK’s Haematological Research Network also reported greater specificity by cancer sub-type: bruising/bleeding and shortness of breath/cough in acute myeloid leukaemia and myelodysplastic syndromes; lymphadenopathy (usually reported as a lump) in lymphoma; joint problems and fractures in myeloma [14]. This study also observed no differences in time-to-diagnosis between those who reported having symptoms cited in the UK Referral Guidelines and those who did not [14]. Further research is recommended, particularly in Australia, using Australian electronic health records, examining the pre-diagnosis signs and symptoms and changes in blood of haematological cancers, to inform existing guidelines and OCPs.

Less than 1 in 5 Australians reported being aware of any blood cancer-related symptoms [38]. Given the increasing incidence of all blood cancers, more investment is urgently needed to raise public awareness of blood cancer symptoms through public information and education campaigns [8]. Campaigns encouraging people to recognise persistent, worsening, or changes that are beyond their usual normal state may prove more effective in promoting timely help-seeking behaviour for such conditions [35]. Applications of electronic safety netting software and risk calculation tools, as well as guiding specialist referral to non-specific symptom pathways [8, 39], could be potential solutions to support effective decision-making and appropriate follow-up for non-specific symptoms. Further research is needed to determine the probability of presenting signs and symptoms, as well as associated blood tests to predict haematological cancers, particularly in Australia, to inform the development of these tools and updates to OCPs. Furthermore, strengthening rapid diagnosis, for example, through the establishment of rapid diagnostic cancer clinics and community diagnostic centres [40, 41], should be considered to facilitate timely diagnosis. However, it is also important not to ignore the benefit of doing inexpensive, timely full blood count (FBC) tests, particularly for blood cancers like leukaemias, for which definitive diagnosis, in general, is within GP practice [34]. Particularly when patients present with a cluster of persistent non-specific symptoms that are deemed a lower threshold, it is worth considering an FBC test rather than delaying it [42].

Research will be useful in demonstrating whether the existing symptom-based multi-cancer screening campaign [43] or blood tests during other regular health check-ups can also be leveraged for early flagging of blood cancer or blood abnormalities in patients and initiating appropriate follow-up and investigation for suspected cases. Any early deviation of blood results (e.g. high platelet count) should also be closely monitored. Emerging research has reported an increased incidence of blood cancer following the detection of high platelet counts [44]. Further research will be pertinent to determine “clusters” of signs, symptoms and blood tests that are strongly indicative of blood cancers to support GPs in detecting suspected cases earlier.

### Strengths and limitations

This research used a large national survey dataset to determine the PHC-haematologist visit intervals and non-haematological referral since patients’ first presentation at PHC. However, an assessment of patient delay and tertiary health care delay is equally important to reduce potential delays along the diagnosis and treatment journey for blood cancer. The PHC-haematologist visit interval comprises of both PHC and tertiary care level intervals. Future research capturing all the delays [7] including the true PHC interval of blood cancer diagnosis in Australia according to the Aarhus definition will be relevant for adapting appropriate intervention strategies to prevent delays and enhance support along the blood cancer diagnosis and treatment journey. We also could not differentiate whether the extended PHC-haematologist visit interval was influenced by the decision of patients or HCPs or time to obtain a specialist appointment. The self-reported time assessment of past blood cancer diagnosis and first relevant health care presentation is subject to some recall bias. Patients who opted to complete the survey may not represent those with short survival following delayed diagnosis, potentially leading to selection bias and underestimating the true burden associated with delayed diagnosis. We also could not assess the type and severity of symptoms that trigger patients to visit a GP. It is also possible that those experiencing referrals to specialists other than haematologists could be due to their existing conditions or risk factors for other diseases. Further research is recommended to determine factors associated with non-haematological referrals to address the associated delay due to such referrals, when appropriate. However, our findings are still relevant for addressing disparities in blood cancer survival and outcomes, as a high proportion of patients experience an extended PHC-haematologist visit interval or non-haematological referrals. We combined low and high-grade NHLs in one group because of the small sample size in some of the NHL subgroups, which is likely to mask the response to some of the aggressive NHLs.

## Conclusions

This is one of the few studies that have shown high proportions of patients experiencing an extended interval between first PHC contact and haematologist visit or non-haematological referrals during blood cancer diagnosis in Australia. Women, regional residents and indolent groups of blood cancers are more likely to experience extended PHC-haematologist intervals. Moreover, younger patients, women, Australian born and those with private health insurance were more likely to experience referrals to a non-haematologist. Education campaigns for raising awareness for patients and HCPs on blood cancer signs and symptoms, as well as enhancing support for PHC at the point of care to facilitate follow-up and blood tests, and expediting time from PHC referral to first haematology appointment, could be some possible options to address these delays and non-haematological referrals. In addition, further evidence on signs and symptoms indicating a lower threshold for using FBCs for suspected blood cancers and factors associated with patient and tertiary level delays will be essential to enhance the blood cancer diagnosis and treatment journey and survival outcomes.

## Supplementary Information

Supplementary Material 1.

## Data Availability

The datasets analysed during the current study are not publicly available due to ethical reasons, but are available from the corresponding author upon reasonable request, subject to the permission of the respective ethics committee.

## Abbreviations

ACT: Australian Capital Territory
aOR: Adjusted odds ratio
HCP: Health care professionals
MDS: Myelodysplastic neoplasm
MM: Multiple myeloma
MMM7: Modified Monash Model 7
MPN: Myeloproliferative neoplasm
NHL: Non-Hodgkin lymphoma
NSW: New South Wales
NT: Northern Territory
OCP: Optimal care pathway
PHC: Primary health care
QLD: Queensland
SA: South Australia
SoTN: State of the Nation
TAS: Tasmania
VIC: Victoria
WA: Western Australia
