# Supplementary material for: Time to haematologist visit and non-haematological referral from primary health care during blood cancer diagnosis – findings from a large national survey in Australia
Source: BMC Cancer. 2026 Feb 17;26:300. doi: 10.1186/s12885-026-15757-1 (PMC12931068; doi:10.1186/s12885-026-15757-1)
Supplement: Supplementary file 1 — Supplementary Material 1. [file 12885_2026_15757_MOESM1_ESM.docx]

**Supplementary Table 1** **Time to haematologist visit category and referral to a haematologist category from primary health care (PHC) by Australian states and territories**

|  | **First PHC presentation to haematologist visit interval** | | | | **PHC referral to a haematologist** | | | |
| --- | --- | --- | --- | --- | --- | --- | --- | --- |
| **States & Territories*** | **< 1-month** | **> 1-month** | **Total** | **p value** | **Referral direct to haematologist** | **Referral to specialists other than haematologists** | **Total** | **p value** |
| ACT (n) | 32 | 13 | 45 | 0.386 | 35 | 8 | 43 | 0.092 |
| % (95% CI) | 71.1 (56.2, 82.5) | 28.9 (17.5, 43.8) |  |  | 81.4 (66.8, 90.5) | 18.6 (9.5, 33.2) |  |  |
| SA | 100 | 31 | 131 |  | 90 | 40 | 130 |  |
|  | 76.3 (68.3, 82.9) | 23.7 (17.1, 31.7) |  |  | 69.2 (60.7, 76.6) | 30.8 (23.4, 39.2) |  |  |
| QLD | 317 | 104 | 421 |  | 318 | 97 | 415 |  |
|  | 75.3 (70.9, 79.2) | 24.7 (20.8, 29.1) |  |  | 76.6 (72.3, 80.5) | 23.4 (19.5, 27.7) |  |  |
| VIC | 247 | 88 | 335 |  | 230 | 100 | 330 |  |
|  | 73.7 (68.7, 78.2) | 26.3 (21.8, 31.2) |  |  | 69.7 (64.5, 74.4) | 30.3 (25.6, 35.5) |  |  |
| WA | 121 | 53 | 174 |  | 132 | 45 | 177 |  |
|  | 69.5 (62.3, 75.9) | 30.5 (24.1, 37.7) |  |  | 74.6 (67.6, 80.4) | 25.4 (19.5, 32.4) |  |  |
| NSW | 378 | 130 | 508 |  | 398 | 114 | 512 |  |
|  | 74.4 (70.4, 78.0) | 25.6 (22.0, 29.6) |  |  | 77.7 (73.9, 81.1) | 22.3 (18.9, 26.1) |  |  |
| NT | 10 | 9 | 19 |  | 15 | 3 | 18 |  |
|  | 52.6 (30.6, 73.7) | 47.4 (26.3, 69.4) |  |  | 83.3 (58.2, 94.7) | 16.7 (5.3, 41.7) |  |  |
| TAS | 84 | 33 | 117 |  | 91 | 25 | 116 |  |
|  | 71.8 (62.9, 79.2) | 28.1 (20.8, 37.1) |  |  | 78.4 (70.0, 85.0) | 21.6 (15.0, 30.0) |  |  |
| **Total** |  |  | 1750 |  |  |  | 1741 |  |

* States & Territories – ACT – Australian Capital Territory, SA – South Australia, QLD – Queensland, VIC – Victoria, WA – Western Australia, NSW – New South Wales, NT – Northern Territory, TAS – Tasmania

**Supplementary Table 2 Multivariable logistic regression analysis on factors associated with extended PHC-haematologist visit interval in New South Wales, Australia**

|  | **Unadjusted** | | | **Adjusted** | | | |
| --- | --- | --- | --- | --- | --- | --- | --- |
|  | **Odds Ratio** | **95% Confidence Interval** | **p value** | | **Odds Ratio** | **95% Confidence Interval** | **p value** |
| **Age groups in years** |  |  |  | |  |  |  |
| 15-34 | Ref |  |  | |  |  |  |
| 35-64 | 1.39 | 0.54, 3.56 | 0.493 | | 0.97 | 0.35, 2.72 | 0.957 |
| >=65 | 1.54 | 0.61, 3.92 | 0.365 | | 1.17 | 0.41, 3.35 | 0.764 |
| **Sex** |  |  |  | |  |  |  |
| Men | Ref |  |  | |  |  |  |
| Women | 1.35 | 0.90, 2.02 | 0.148 | | 1.46 | 0.94, 2.28 | 0.095 |
| **Residence** |  |  |  | |  |  |  |
| Metro | Ref |  |  | |  |  |  |
| Regional | 2.10 | 1.32, 3.33 | 0.002 | | 1.95 | 1.19, 3.20 | 0.008 |
| **Country of birth** |  |  |  | |  |  |  |
| Australia | Ref |  |  | |  |  |  |
| Overseas | 0.79 | 0.60, 1.03 | 0.076 | | 0.67 | 0.38, 1.19 | 0.170 |
| **Income** |  |  |  | |  |  |  |
| <$50k | Ref |  |  | |  |  |  |
| >=$50k | 1.09 | 0.71, 1.68 | 0.699 | | 1.31 | 0.79, 2.17 | 0.289 |
| Undisclosed | 0.74 | 0.40, 1.36 | 0.329 | | 0.72 | 0.37, 1.41 | 0.343 |
| **Private insurance** |  |  |  | |  |  |  |
| No | Ref |  |  | |  |  |  |
| Yes | 0.95 | 0.77, 1.17 | 0.620 | | 0.95 | 0.76, 1.20 | 0.673 |
| **Blood cancer^ diagnosis** |  |  |  | |  |  |  |
| Acute Leukaemia | Ref |  |  | |  |  |  |
| Chronic Leukaemia | 4.11 | 1.97, 8.59 | 0.000 | | 3.87 | 1.8, 8.29 | 0.001 |
| Hodgkin Lymphoma | 2.06 | 0.65, 6.54 | 0.219 | | 1.96 | 0.6, 6.42 | 0.266 |
| MDS | 5.22 | 1.92, 14.20 | 0.001 | | 5.26 | 1.88, 14.68 | 0.002 |
| MM | 2.41 | 1.10, 5.27 | 0.028 | | 2.42 | 1.07, 5.45 | 0.033 |
| MPN | 4.10 | 1.59, 10.56 | 0.003 | | 4.09 | 1.53, 10.94 | 0.005 |
| NHL | 3.04 | 1.51, 6.14 | 0.002 | | 2.92 | 1.41, 6.05 | 0.004 |

^ blood cancer – MDS – Myelodysplastic neoplasm, MM – Multiple myeloma, MPN – Myeloproliferative neoplasm, NHL- Non-Hodgkin Lymphoma

**Supplementary Table 3 Multivariable logistic regression analysis on factors associated with PHC referral to specialists other than haematologists during blood cancer diagnosis in New South Wales, Australia**

|  | **Unadjusted** | | | **Adjusted** | | |
| --- | --- | --- | --- | --- | --- | --- |
|  | **Odds Ratio** | **95% Confidence Interval** | **p value** | **Odds Ratio** | **95% Confidence Interval** | **p value** |
| **Age groups in years** |  |  |  |  |  |  |
| 15-34 | Ref |  |  |  |  |  |
| 35-64 | 0.69 | 0.30, 1.59 | 0.382 | 0.49 | 0.19, 1.26 | 0.137 |
| >=65 | 0.74 | 0.33, 1.70 | 0.481 | 0.55 | 0.2, 1.46 | 0.226 |
| **Sex** |  |  |  |  |  |  |
| Men | Ref |  |  |  |  |  |
| Women | 1.63 | 1.06, 2.50 | 0.027 | 1.74 | 1.09, 2.78 | 0.021 |
| **Residence** |  |  |  |  |  |  |
| Metro | Ref |  |  |  |  |  |
| Regional | 1.01 | 0.65, 1.56 | 0.967 | 0.89 | 0.54, 1.45 | 0.632 |
| **Country of birth** |  |  |  |  |  |  |
| Australia | Ref |  |  |  |  |  |
| Overseas | 0.86 | 0.66, 1.12 | 0.269 | 0.70 | 0.39, 1.26 | 0.237 |
| **Income** |  |  |  |  |  |  |
| < $50k | Ref |  |  |  |  |  |
| >=$50k | 0.90 | 0.57, 1.42 | 0.650 | 1.01 | 0.6, 1.72 | 0.963 |
| Undisclosed | 0.76 | 0.41, 1.41 | 0.379 | 0.79 | 0.4, 1.55 | 0.491 |
| **Private insurance** |  |  |  |  |  |  |
| No | Ref |  |  |  |  |  |
| Yes | 1.08 | 0.87, 1.35 | 0.491 | 1.16 | 0.9, 1.49 | 0.241 |
| **Blood cancer^ diagnosis** |  |  |  |  |  |  |
| Acute Leukaemia | Ref |  |  |  |  |  |
| Chronic Leukaemia | 1.54 | 0.68, 3.48 | 0.301 | 1.65 | 0.71, 3.84 | 0.247 |
| Hodgkin Lymphoma | 4.27 | 1.5, 12.16 | 0.007 | 4.93 | 1.65, 14.74 | 0.004 |
| MDS | 1.45 | 0.43, 4.94 | 0.548 | 1.57 | 0.45, 5.49 | 0.481 |
| MM | 2.06 | 0.93, 4.55 | 0.075 | 2.39 | 1.04, 5.49 | 0.040 |
| MPN | 1.14 | 0.34, 3.82 | 0.828 | 1.12 | 0.33, 3.84 | 0.859 |
| NHL | 4.76 | 2.39, 9.5 | 0.000 | 5.79 | 2.78, 12.05 | 0.000 |

^ blood cancer – MDS – Myelodysplastic neoplasm, MM – Multiple myeloma, MPN – Myeloproliferative neoplasm, NHL- Non-Hodgkin Lymphoma
